# Stretching of Putative Mechanoreceptors in the Inferior Tarsal Muscle Regulates Tonic and Clonic Reflex Contractions of Slow-Twitch Fibers in the Palpebral Orbicularis Oculi Muscle Causing Apraxia of Eyelid Opening: A Case Series

**DOI:** 10.7759/cureus.62450

**Published:** 2024-06-15

**Authors:** Kiyoshi Matsuo, Ai Kaneko

**Affiliations:** 1 Plastic and Oculoplastic Surgery, Matsuo Plastic and Oculoplastic Surgery Clinic, Hamamatsu, JPN; 2 Plastic and Reconstructive Surgery, Shinshu University School of Medicine, Matsumoto, JPN

**Keywords:** rostral locus coeruleus, mesencephalic trigeminal nucleus, retractor disinsertion, lower eyelid retractor, global layer of inferior rectus muscle, microsaccades, proprioceptor, inferior tarsal muscle, palpebral orbicularis oculi muscle, apraxia of eyelid opening

## Abstract

The levator palpebrae superioris muscle (LPSM) and facial muscles comprise both fast-twitch fibers (FTFs) and slow-twitch fibers (STFs). Still, they lack the muscle spindles to induce reflex contractions of STFs. Because reflex contractions of STFs in the LPSM and frontalis muscle, which are the major eyelid opening muscles, are induced by stretching of mechanoreceptors in the superior tarsal muscle, those in the palpebral orbicularis oculi muscle (POOM), which is the major eyelid closing muscle, should not be induced by stretching of the same proprioceptors but instead induced by the proprioceptors in the vicinity of the POOM. Apraxia of eyelid opening (AEO) after eyelid closure might be caused by prolonged POOM contraction. Most patients with AEO tend to stretch the upper and lower eyelids by applying contact lenses and eyedrops to disinsert the aponeurosis and retractor from the tarsi. They taught us that pulling down or raising the lower eyelid decreased or increased involuntary contraction of the POOM, which relieved or worsened AEO, respectively. Then, they asked us to have the lower eyelid lowered and the upper eyelid raised surgically. Whenever the upper eyelid is opened by contractions of the LPSM with the global layer of superior rectus muscle (GLSRM), the lower eyelid is concomitantly opened by contractions of the global layer of inferior rectus muscle (GLIRM), which counteracts the contraction of the GLSRM to maintain the visual axis. We hypothesized that patients with retractor disinsertion raise the lower eyelid by eyelid closure to stretch putative mechanoreceptors in the inferior tarsal muscle (ITM), which induces prolonged tonic and clonic reflex contractions of STFs in the POOM, resulting in AEO. To retrospectively verify the hypothesis, we report five cases with AEO.

In the first case, AEO was induced by tight eyelid closure but was prevented by pulling down the lower eyelid during eyelid closure. Surgery to reinsert retractors into the tarsi cured AEO. In the second case, the patient sustained both severe aponeurosis-disinserted blepharoptosis and AEO. In this patient, the first surgery to reinsert aponeuroses to the the tarsi cured AEO, but a tight eyelid closure induced prolonged POOM contraction. The second surgery conducted to reinsert the retractors to the tarsi cured AEO. In the third case, with the entire eyelid AEO, surgery done to reinsert the retractors to the tarsi almost cured the entire eyelid AEO. In the fourth case, an increased clonic contraction of the POOM on the right eyelid after a tight eyelid closure was relieved by 4% lidocaine instillation to anesthetize the ITM. In the fifth case, downgaze induced clonic reflex contraction of the right POOM because of the right retractor disinsertion. Thus, prolonged tonic and clonic reflex contractions of STFs in the POOM appeared to be regulated by enhanced stretching of putative mechanoreceptors in the ITM in patients with retractor disinsertion due to increased contractions and microsaccades of FTFs in the GLIRM. Because reflex contractions of STFs in the POOM by stretching of putative mechanoreceptors in the ITM might essentially attach the upper and lower eyelids to the globe, AEO might simply be the increased reflex contraction of the POOM.

## Introduction

The levator palpebrae superioris muscle (LPSM) and the facial muscles comprise both fast-twitch fibers (FTFs) and slow-twitch fibers (STFs). However, the muscle spindles that induce reflex contractions of STFs are absent [1-3]. The superior tarsal muscle (STM) originates from the undersurface of the LPSM, which interdigitates with the skeletal and smooth muscle fibers [4]. The STM connects to the upper surface of the tarsus. It has a sparse innervation of unmyelinated sympathetic nerve fibers efferently and is abundantly innervated by myelinated proprioceptive nerve fibers in a palisade arrangement (which function as mechanoreceptors in the STM) afferently [5,6]. The proprioceptive nerve fibers reach the mesencephalic trigeminal nucleus (MTN) and scatteredly connect to the neurons of the rostral locus coeruleus (RLC) through gap junctions [7].

Microsaccades are a kind of fixational eye movement. Involuntary, small contractions of the extraocular muscles of the globe at a frequency of 1-3 Hz displace the globe. This ensures that the vision does not fade during fixation because of neural adaptation [8] and that the proprioception evoked by stretching the mechanoreceptors in the STM does not fade during eyelid opening. Voluntary contractions and microsaccades of FTFs in the LPSM with the global layer of superior rectus muscle (GLSRM) [9,10] stretch the mechanoreceptors in the STM to continually induce trigeminal proprioceptive activation (TPA). TPA regulates not only the reflex contractions of STFs in the LPSM, frontalis muscle, and orbital orbicularis oculi muscle (OOOM) for eye-eyelid-eyebrow coordinated movements [11-14] but also the physiological arousal with prefrontal blood flow increases and sympathetic activation such as palmar sweating via the MTN and RLC [15]. Because reflex contractions of STFs in the LPSM (the major eyelid opening muscle) are induced by stretching of mechanoreceptors in the STM, those in the palpebral orbicularis oculi muscle (POOM) (the major eyelid closing muscle) should not be caused by stretching of the same proprioceptors but instead by the proprioceptors in the vicinity of the POOM.

Apraxia of eyelid opening (AEO) after eyelid closure might be caused by prolonged POOM contraction [16]. Most patients with AEO have habitually stretched their upper and lower eyelids due to applying contact lenses and eyedrops to disinsert aponeurosis and retractor from the tarsi [17]. These patients experience trembling eyelids, which could be induced by small clonic contractions of the POOMs even when the eyelids are lightly closed. They taught us that pulling down or raising the lower eyelid decreased or increased the involuntary contraction of the POOMs, which relieved or worsened AEO, respectively. Then, they asked us to have the lower eyelid lowered and the upper eyelid raised surgically. Whenever the upper eyelid is opened by contractions of the LPSM with the GLSRM, the lower eyelid is concomitantly opened by contractions of the global layer of inferior rectus muscle (GLIRM), which counteracts the contraction of the GLSRM to maintain the visual axis. Thus, we hypothesized that patients with retractor disinsertion raise the lower eyelid by eyelid closing to stretch putative mechanoreceptors in the inferior tarsal muscle (ITM) [18], which induce prolonged tonic and clonic reflex contractions of STFs in the POOM, causing AEO.

To retrospectively verify the hypothesis, we report five cases with AEO. This report was previously presented as two oral speeches at the 62nd Annual Meeting of the Japan Society of Plastic and Reconstructive Surgery on May 15-17th, 2019.

## Case presentation

**Figure 1:**
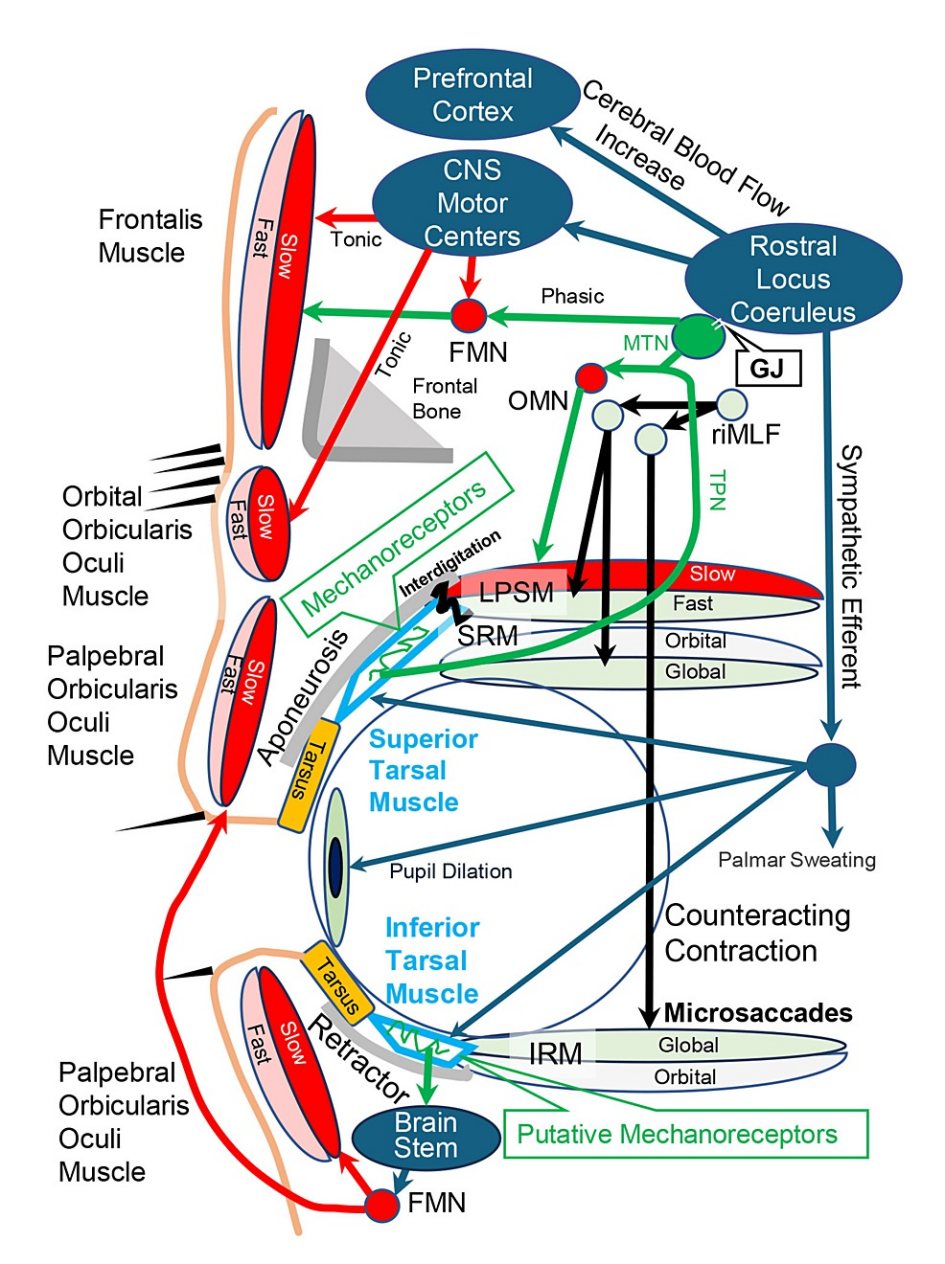
Neuroanatomy of the induced reflex contractions of STFs in the levator palpebrae superioris, frontalis, orbital orbicularis oculi, and POOMs The levator palpebrae superioris muscle (LPSM) interdigitates (Interdigitation) with the STM. Voluntary contractions of FTFs (Fast) in the LPSM and the global layer (Global) of the superior rectus (SRM) via the rostral interstitial nucleus of the medial longitudinal fasciculus (riMLF) and the oculomotor neurons (OMN) stretch the mechanoreceptors in the STM, which induce phasic reflex contractions (Phasic) of STFs (Slow) in the LPSM and frontalis muscle via the mesencephalic trigeminal nucleus (MTN), oculomotor neurons (OMN), and frontalis motor neurons (FMN). Because the MTN connects the RLC through gap junctions (GJ), the RLC projections to CNS motor centers induce tonic (Tonic) reflex contractions of STFs in the frontalis muscle and OOOM. Counteracting contraction and microsaccades (Microsaccades) of FTFs in the global layer of the inferior rectus muscle (IRM) stretch the putative mechanoreceptors in the ITM to induce tonic and clonic reflex contraction of STFs in the POOM via the brainstem. FTFs: fast-twitch fibers, STFs: slow-twitch fibers, POOM: palpebral orbicularis oculi muscle, ITM: inferior tarsal muscle, STM: superior tarsal muscle, RLC: rostral locus coeruleus, OOOM: orbital orbicularis oculi muscle, TPN: trigeminal proprioceptive nerve Image Credit: Kiyoshi Matsuo and Ai Kaneko

Figure 1 presents the neuroanatomy of the induced reflex contractions of STFs in the LPSM, frontalis muscle, and OOOM caused by stretching of the mechanoreceptors in the STM and the induced tonic and clonic reflex contractions of STFs in the POOM caused by stretching of the putative mechanoreceptors in the ITM.

Case 1

**Figure 2:**
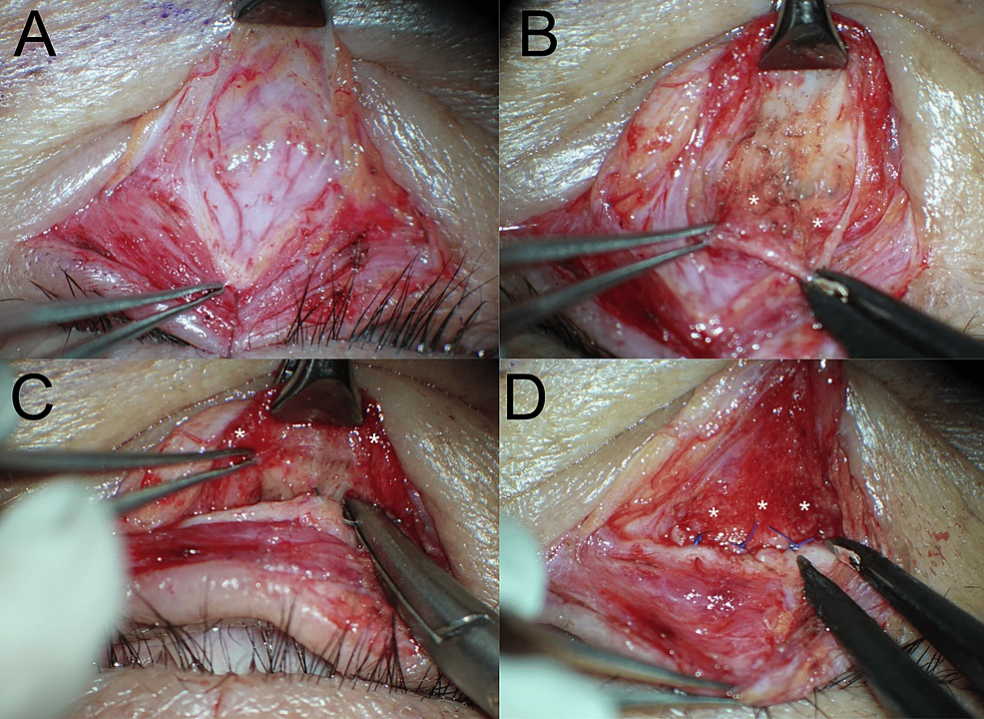
Surgical steps to reinsert the retractor to the tarsus (A) Through the subciliary skin incision, the conjunctiva palpebrae are dissected from the lower eyelid retractor and the ITM. The firm retractor and ITM are not dissected from these tissues because the slippage between the firm lower eyelid retractor and the tarsus is larger than expected. (B) Based on our experience, the ITM consists of a flat contractile portion with the conjunctiva palpebrae and two bulbar proprioceptor portions with the fornix. Around the fornix, the firm lower eyelid retractor is dissected from under the flat contractile portion of the ITM. Two forceps pinch the fornix. The two white asterisks indicate the medial and central bulbar portions of the ITM, confirmed by intraoperative downgazing. The bulbar portions probably contain the mechanoreceptors and connect the global layer of the inferior rectus muscle with interdigitations similar to the interdigitations between the STM and the LPSM. (C, D) The distal, firm retractor is reinserted into the tarsus using a 7-0 nonabsorbable thread. The white asterisks indicate the contractile portion of the ITM. LPSM: levator palpebrae superioris muscle, ITM: inferior tarsal muscle, STM: superior tarsal muscle

A 63-year-old male presented with AEO after tight eyelid closure (Video 1). His disinserted aponeuroses were already fixed to the tarsi. AEO was induced by tight eyelid closure but was prevented by pulling down the lower eyelid during eyelid closure. Digitally raising the bilateral lower eyelid margins increased the contraction of the POOM. Unilateral eyelid closure induced prolonged tonic and clonic contractions of the bilateral POOMs with ipsilateral dominance. After the surgery, which was done to fix the disinserted retractors to the tarsi (Figure 2), a tight eyelid closure did not increase the reflex contraction of the POOM, hence curing AEO (Video 2).

**Video 1:** Preoperative eyelid movement (case 1) Although an upgaze that stretches the mechanoreceptors in the STM does not induce reflex contractions in any part of the orbicularis oculi muscles, eyelid closure by raising the lower eyelid margin to stretch the putative mechanoreceptors in the ITM induces reflex tonic and clonic contractions of the POOMs. The unilateral eyelid closure induces reflex tonic and clonic contractions of the bilateral palpebral orbicularis muscles with ipsilateral dominance implying that the neural circuits for the reflex arch might be routed through the brainstem. POOMs: palpebral orbicularis oculi muscles, ITM: inferior tarsal muscle, STM: superior tarsal muscle

**Video 2:** Ten months postoperative eyelid movement (case 1) Bilateral and unilateral eyelid closures do not induce reflex tonic and clonic contractions of the POOMs, hence curing the AEO. POOMs: palpebral orbicularis oculi muscle, AEO: apraxia of eyelid opening

Case 2

A 55-year-old female presented with both severe aponeurosis-disinserted blepharoptosis and AEO (Video 3). Although the first surgery conducted to reinsert aponeuroses to the tarsi almost cured the AEO, a tight eyelid closure continued to induce prolonged clonic contractions of the POOMs (Video 4). The second surgery conducted to reinsert retractors in the tarsi cured AEO (Video 5).

**Video 3:** Eyelid movement before aponeurosis reinsertion surgery (case 2) The patient cannot open her eyelids without digitally pulling down the lower eyelids.

**Video 4:** Eyelid movement after aponeurosis reinsertion surgery and before retractor reinsertion surgery (case 2) The patient can open the eyelids while upgazing and after light eyelid closure. However, a tight eyelid closure slightly increases reflex tonic and clonic contractions of the POOMs. POOMs: palpebral orbicularis oculi muscles

**Video 5:** Eyelid movement after retractor reinsertion surgery (case 2) Tight eyelid closure does not increase reflex tonic and clonic contractions of the POOMs, hence curing the AEO. POOMs: palpebral orbicularis oculi muscles, AEO: apraxia of eyelid opening

Case 3

A 67-year-old female presented with increased contraction of the entire POOMs, which induced entire eyelid AEO after tight eyelid closure. Upgaze did not increase contractions of the POOMs, but bilateral tight eyelid closure increased tonic and clonic contractions of the POOMs (Video 6). Based on the patient’s wishes, the disinserted retractors were fixed to the lower tarsi. One month after the surgery, increased contractions of the POOMs while opening the eyelid and the entire eyelid AOE after tight eyelid closure were satisfactorily cured (Video 7).

**Video 6:** Preoperative eyelid movement (case 3) Reflex contractions of the POOMs, consisting of the pretarsal and presetptal orbicularis oculi muscles, are increased. POOMs: palpebral orbicularis oculi muscles

**Video 7:** One month postoperative eyelid movement (case 3) Retractor reinsertion surgery decreased reflex contractions of the POOMs while primary gazing, upward gazing, and after eyelid closure. POOMs: palpebral orbicularis oculi muscles

Case 4

A 71-year-old female presented with asymmetrically increased POOM contractions. Bilaterally tight eyelid closure asymmetrically increased tonic and clonic contractions of the POOMs, but not to the extent of eyelid closure like AEO (Video 8). After 4% lidocaine instillation to the right lower fornix for anesthetizing the putative mechanoreceptors in the right ITM, tonic and clonic contractions of the right POOM before and after bilateral tight eyelid closure decreased (Video 9).

**Video 8:** Eyelid movement before 4% lidocaine instillation to the right lower fornix (case 4) Tight eyelid closure increased reflex tonic and clonic contractions of the POOMs, narrowing the palpebral fissure. POOMs: palpebral orbicularis oculi muscles

**Video 9:** Eyelid movement after 4% lidocaine instillation to the right lower fornix (case 4) Reflex contraction of the right POOM decreased, widening the palpebral fissure. However, strong eyelid opening with increasing counteracting contractions and microsaccades of the global layer of the inferior rectus muscle and tight eyelid closure enhanced the putative mechanoreceptor stretching and induced clonic contractions, such as myokymia. POOM: palpebral orbicularis oculi muscle

Case 5

A 45-year-old female with right retractor disinsertion presented with voluntarily induced clonic contraction of the right POOM (such as myokymia). Her disinserted aponeuroses were already fixed to the tarsi. Strong downgazing with enhanced stretching of the putative mechanoreceptors in the ITM due to increased contraction and microsaccades of the GLIRM induced prolonged clonic contraction of the POOM in the right upper and lower eyelids (Video 10).

**Video 10:** Strong downgazing to enhance the putative mechanoreceptor stretching causes clonic contraction of the right POOM (case 5) After aponeurosis reinsertion surgery, the right lower eyelid retractor is more reinserted than the left one, and hence, the patient’s right palpebral fissure is narrower than the left one. This is due to the increased contraction of the POOM in the right eyelid. Strong downgazing to enhance the putative mechanoreceptor stretching induces prolonged tonic and clonic contraction of the right POOM, similar to myokymia. POOM: palpebral orbicularis oculi muscle

## Discussion

Enhanced stretching of the putative mechanoreceptors in the ITM by tight eyelid closure, digitally raising the lower eyelid margin, and downgazing appeared to increase prolonged reflex tonic and clonic contractions of STFs in the POOMs, whereas reduced stretching of the putative mechanoreceptors in the ITM by digitally pulling down the lower eyelid margin, surgical reinsertion of the lower eyelid retractor to the tarsus, and anesthesia of the ITM decreased the reflex contraction of STFs in the POOMs. Thus, prolonged tonic and clonic reflex contractions of STFs in the POOM appeared to be induced by enhanced stretching of the putative mechanoreceptors in the ITM in patients with retractor disinsertion (as shown in case 1). Reflex contractions of STFs in the POOM caused by stretching of the putative mechanoreceptors in the ITM might essentially attach the upper and lower eyelids to the globe. Increased reflex contraction of the POOM for closing the eyelid could overcome the impaired retractile force of the upper eyelid in patients with aponeurosis disinsertion, resulting in AEO (as shown in case 2). Because stretching the putative mechanoreceptors in the ITM induced the increased reflex contraction of the pretarsal and preseptal orbicularis oculi muscles, such as the entire eyelid AEO (as shown in cases 3 and 5), we defined the increased reflex contraction of the POOM as causing AOE. Clonic reflex contractions of STFs in the POOM might be induced by stretching of the putative mechanoreceptors in the ITM, probably due to microsaccades of the FTFs in the GLIRM (as shown in cases 4 and 5). Because microsaccade velocity was increased for arousal regulation [19], the clonic rippling movements in the POOM, for example, in cases of myokymia, might be induced by the same reflex arch. For stress management, stretching of the mechanoreceptors in the STM by increased contractions of the LPSM with the GLSRM was enhanced to regulate a brainstem arousal mechanism [15]. Subsequently, increased counteracting contractions and microsaccades of the FTFs in the GLIRM might increase the clonic reflex contraction of the POOM, resulting in myokymia.

People who habitually stretch their upper and lower eyelids, such as by applying contact lenses and eyedrops, commonly sustain the disinsertion of both the retractor and the aponeurosis from the tarsi. Because they increase voluntary contractions of the LPSM with the GLSRM, the counteracting contraction of the GLIRM is increased to stretch the putative mechanoreceptors in the ITM, increasing the reflex contraction of the POOM and narrowing the palpebral fissure. This increased contraction of the POOM might enhance the stretching of the mechanoreceptors in the STM, in turn affecting the brainstem arousal mechanism [15] and CNS motor centers via the MTN and RLC [20].

## Conclusions

The five cases indicated that AEO might simply be an increased reflex contraction of the POOM. We explored the proprioceptors outside of the LPSM and facial muscles, which induced reflex contractions of the corresponding STFs instead of the muscle spindles. The mechanoreceptors in the STM regulated reflex contractions of STFs in the LPSM, frontalis muscle, and OOOM, whereas the putative mechanoreceptors in the ITM regulated reflex contractions of the STFs in the POOM. The putative mechanoreceptors in the ITM functionally exist; however, further neuroanatomical and neurophysiological studies on mechanoreceptors are needed.
